# Bioinspired integrated triboelectric electronic tongue

**DOI:** 10.1038/s41378-024-00690-9

**Published:** 2024-05-08

**Authors:** Jiaming Liu, Jingui Qian, Murtazt Adil, Yali Bi, Haoyi Wu, Xuefeng Hu, Zuankai Wang, Wei Zhang

**Affiliations:** 1 Anhui Province Key Laboratory of Measuring Theory and Precision Instruments, School of Instrumental Science and Optoelectronics Engineering, Hefei University of Technology, 230009 Hefei, Anhui China; 2 School of Physics and Optoelectronic Engineering, Guangdong University of Technology, 510006 Guangzhou, Guangdong China; 3 Department of Mechanical Engineering, The Hong Kong Polytechnical University, Hong Kong SAR, China

**Keywords:** Biosensors, Electrical and electronic engineering

## Abstract

An electronic tongue (E-tongue) comprises a series of sensors that simulate human perception of taste and embedded artificial intelligence (AI) for data analysis and recognition. Traditional E-tongues based on electrochemical methods suffer from a bulky size and require larger sample volumes and extra power sources, limiting their applications in in vivo medical diagnosis and analytical chemistry. Inspired by the mechanics of the human tongue, triboelectric components have been incorporated into E-tongue platforms to overcome these limitations. In this study, an integrated multichannel triboelectric bioinspired E-tongue (TBIET) device was developed on a single glass slide chip to improve the device’s taste classification accuracy by utilizing numerous sensory signals. The detection capability of the TBIET was further validated using various test samples, including representative human body, environmental, and beverage samples. The TBIET achieved a remarkably high classification accuracy. For instance, chemical solutions showed 100% identification accuracy, environmental samples reached 98.3% accuracy, and four typical teas demonstrated 97.0% accuracy. Additionally, the classification accuracy of NaCl solutions with five different concentrations reached 96.9%. The innovative TBIET exhibits a remarkable capacity to detect and analyze droplets with ultrahigh sensitivity to their electrical properties. Moreover, it offers a high degree of reliability in accurately detecting and analyzing various liquid samples within a short timeframe. The development of a self-powered portable triboelectric E-tongue prototype is a notable advancement in the field and is one that can greatly enhance the feasibility of rapid on-site detection of liquid samples in various settings.

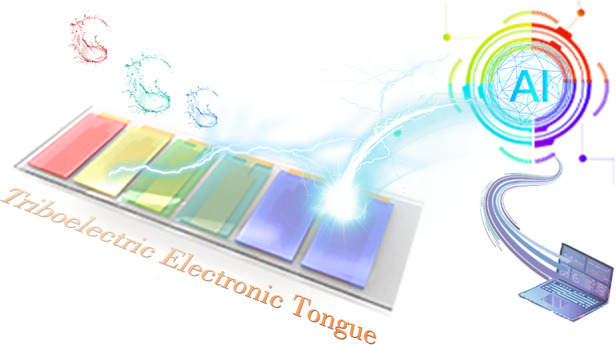

## Introduction

The sense of taste is an essential biological function that enables humans to perceive a wide range of flavors. This ability to discern a wide range of tastes with relatively small few taste buds is facilitated by the integration of signals generated at taste buds and subsequent analysis and recognition by neural networks inside the brain^1^. The electronic tongue (E-tongue) utilizes nonselective sensors to replicate taste receptors and employs artificial intelligence (AI) algorithms to simulate the analytical processes of the brain^2^ and was developed with inspiration from mechanisms underlying natural taste perception. In contrast to natural taste, the concept of “artificial taste” demonstrates a wide range of responses and offers the benefits of objectivity and reproducibility^2^. These prominent features have facilitated the widespread implementation of E-tongues in diverse sectors. For instance, in the food industry, E-tongues are used to differentiate the quality of red wine^3^ and beer^4^ as well as to identify the production regions of rice^5^ and ginger^6^. In the field of environmental monitoring, E-tongues are employed to detect heavy metal ions^7^. Similarly, in the pharmaceutical industry, these devices are used to adjust the taste of medications^8^.

Traditional E-tongues are currently being developed using electrochemical methods, which include voltammetry^9,10^ and potentiometry^2^. In these processes, the sensing electrodes of E-tongues need to be immersed in a liquid sample, often requiring more than 15 mL of sample, and they need to be continuously powered by external energy sources. Additionally, the bulky nature of the instrument requires a considerable amount of space, as observed with an industrial E-tongue (TS-5000Z2) with dimensions of 470 × 530 ×. 510 mm and a weight of 26 kg. Consequently, the testing of E-tongue technology is conducted mainly within laboratory settings, utilizing commercially available products^2,9^. This limitation severely hinders its feasibility for portable, on-site, or real-time applications. Therefore, it is crucial to explore novel detection techniques and develop miniaturized devices to advance the modernization of E-tongue technology. The triboelectric nanogenerator (TENG) was first introduced at Georgia Tech in 2012^11^. It represents a novel approach for energy harvesting and self-powered sensing, which is characterized by its cost-effectiveness, simple fabrication process, and notable efficiency in converting kinetic energy into electrical energy^12–15^. In addition, solid‒solid TENGs have been employed as sensors for detecting wind speed and direction^16^, vector motion monitoring^17^, and 3D tactile sensing^18^. Furthermore, multichannel TENGs can be integrated with AI technology for material identification^19^ and distinguishing human gestures^20^.

Recently, based on the principle of solid‒liquid contact triboelectrification, researchers have developed TENGs as liquid energy harvesters^21–24^ and have further utilized these sensors for droplet detection, wherein features of liquid samples are extracted from the generated electrical signal^25–28^. Triboelectric-based liquid sensors have demonstrated promising potential for applications across various fields^29,30^. Hu et al. developed prototypes of a drainage bottle droplet sensor and a smart intravenous injection monitor to enable real-time monitoring of clinical drainage operations and intravenous infusion^31^. Liu et al. developed a highly sensitive and self-powered acid rain sensor using a doping technique, and their sensor facilitated the real-time detection of acid rain in actual environments^32^. Our research has been motivated by these prior investigations, leading us to propose a novel approach for evaluating the effectiveness of E-tongue technology. The integration of the triboelectric principle into the E-tongue may yield significant insights and contributions. In contrast to the electrochemical-based E-tongue system, the triboelectric-based E-tongue system is considered a pioneering advancement with notable technological advantages, including the capability of self-powered sensing^33^, the utilization of an ultrasmall sample volume (e.g., ~3 μL) per analysis^33^, the exploitation of the electrical properties of the liquid sample derived from natural bionics, and the miniaturized and portable packaging of the device. In a recent study, Dr. Wang and his team introduced a new triboelectric E-tongue^34^, which employs a range of polymer materials to construct independent triboelectric electronic nose gauging cells, allowing the measurement of the triboelectric effect of droplets independently within different units. Although this method effectively provides efficient droplet triboelectric responses, it is time-consuming and labor-intensive, rendering it less suitable for practical applications. Furthermore, other factors, such as the flow state and adsorption capacity when different polymer materials and droplets come into contact, significantly affect the classification accuracy. Therefore, the development of a multichannel triboelectric bionic E-tongue for synchronous measurements is a pivotal step toward the realization of practical applications using triboelectric E-tongues.

**Fig. 1:**
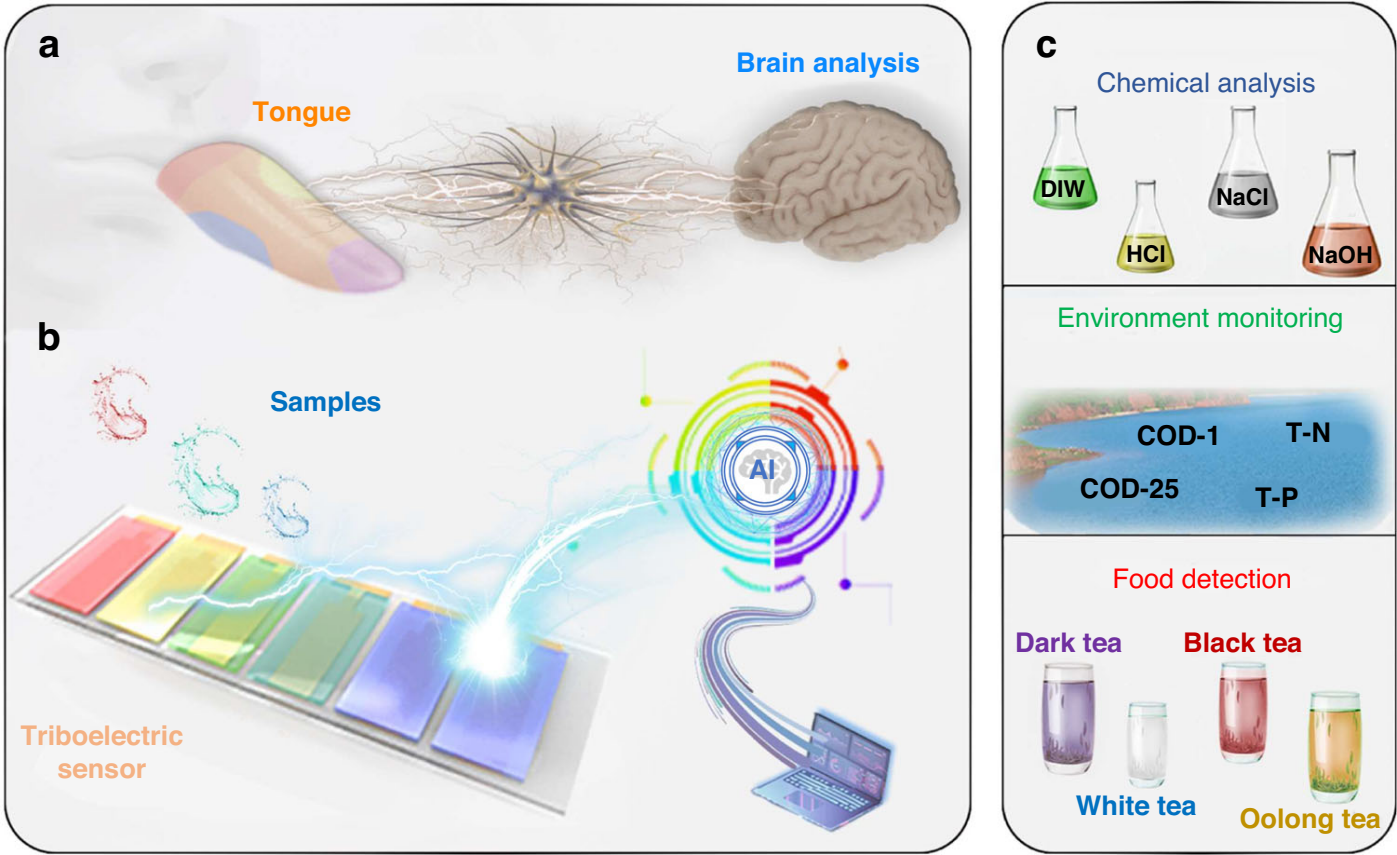
Conceptual diagram of the TBIET device combined with AI for droplet detection. **a** Functional bionics of human taste perception and data analysis. **b** Structural diagram of an artificial taste system using a triboelectric sensor array. **c** Differential application of the TBIET

As depicted in Fig. 1, a conceptually designed triboelectric electronic tongue is presented and compared to a natural tongue. There are five types of taste cells^1^ located on the surface of the tongue. The signals collected by these cells upon tasting a sample are transmitted to the brain through nervous tissue. These signals are then combined into a map and subjected to analysis to ascertain the taste profile of the sample. Motivated by this human taste perception system, we initially developed an on-chip triboelectric bioinspired E-tongue (TBIET) device, which was combined with AI to create an ‘artificial taste’ system that is portable, self-powered, and capable of lossless detection. To evaluate the classification capabilities of the TBIET across various domains, a series of tests were conducted using samples from the chemical, environmental, and food sectors. The experimental findings exhibit a high classification accuracy ( ≥ 97.0%). Moreover, the sample concentration classification accuracy (e.g., NaCl solutions) of TBIET reached 96.9%. These results indicate that the TBIET system is viable for specific application scenarios. The development of the proposed TBIET may afford new possibilities in next-generation self-powered liquid sensing technologies.

## Materials and methods

### Liquid sample preparation

To evaluate the classification capability of the TBIET system for different types of samples, three types of liquid samples were prepared to evaluate the classification capabilities of the TBIET device. Chemical samples (DIW, HCl solution, NaOH solution, and NaCl solution) were purchased from Sinopharm Chemical Reagent Co. and diluted to a concentration of 1 mol/L. Environmental samples (COD-1, COD-25, and T-N at 1 mg/L and T-P at 1 mg/L) were purchased from China Grinm Group Corporation Limited and diluted to their respective concentrations. Food samples (white tea, black tea, dark tea, and oolong tea) were purchased from a public market. These samples were then weighed to 0.3 g and subsequently mixed with 30 mL of DI water.

To assess the classification capability of the TBIET system for samples of the same type but at different concentrations, we prepared five NaCl solutions with concentrations of 0 (DIW), 0.05, 0.1, 1.0, and 5.4 (saturated) mol/L.

### Fabrication of the TBIET device

**Fig. 2:**
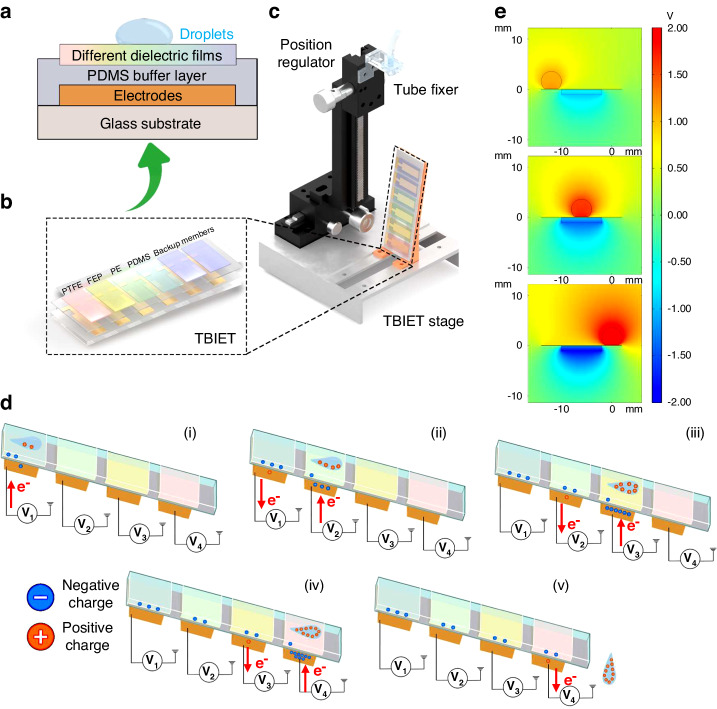
Structural schematic diagram and working mechanism of the TBIET device. **a** A cross-sectional review showing four layers of the TBIET. **b** A three-dimensional structure of the TBIET device. **c** A position adjustable support stage for fixing the TBIET and dropper tube. **d** Work principle of the TBIET. **e** The potential distribution across the electrodes and droplets was simulated using COMSOL Multiphysics

The TBIET device consists of four distinct layers, as shown in Fig. 2a, b: a glass substrate, a metal electrode, a polydimethylsiloxane (PDMS) buffer layer, and a triboelectric film. The size of the glass substrate was 75*25 mm. A thick layer (300 nm) of aluminum electrode was deposited onto the surface of the substrate. The formation of the electrode film pattern was achieved by employing a metal mask to cover the substrate during the magnetron sputter deposition process. The width and spacing of each electrode were 8 and 6 mm, respectively. To prevent penetration of the testing liquid between the dielectric films and the electrodes, an 80 μm thick PDMS buffer layer was spin-coated onto the electrode layer. Then, each triboelectric film was attached to the PDMS buffer layer in its liquid state. Finally, the device underwent an air drying process for 72 h to facilitate the curing of the PDMS buffer layer. This process led to the fixation of the dielectric films onto the PDMS layer. The triboelectric materials selected for this study were polytetrafluoroethylene (PTFE), fluorinated ethylene propylene (FEP), polyethylene (PE) with a thickness of 100 nm, and spin-coated PDMS. The films deposited on the fifth and sixth electrodes were considered supplementary components.

### Characterization of the TBIET device

The TBIET device was mounted on a 3D-printed bracket at an 80° angle (with respect to the horizontal plane), as shown in Fig. 2c. Two graduated sliders were used to adjust the spatial orientation of the dropper relative to the device. To ensure consistent separation between the first droplet and the triboelectric polymer film, the dropper tube was positioned approximately 6 cm from the 4th electrode of the TBIET. The four electrodes were connected to an oscilloscope (RTB2004, Rohde Schwarz) to establish a 4-channel triboelectric sensor array. Optical images depicting these configurations are presented in S1. To process the output signal from the TBIET, three algorithms, linear discriminant analysis (LDA)^35^, support vector machine (SVM)^36^, and random forest (RF)^37^, were used to analyze the acquired data. These algorithms were implemented in Python (version 3.8, Python Software Foundation, Delaware, USA) using the machine learning library scikit-learn (version 1.0.2).

## Work mechanism of the TBIET device

The operational procedure of the TBIET device comprises three distinct steps: initial, unsaturated, and saturated stages^38^. In the initial stage, the system surface is uncharged. When the droplet falls on the surface of the TBIET, charge accumulation begins, indicating an unsaturated stage. After the surface charges are saturated, the entire system transitions into the saturation phase^38^. The maintenance of a stable and consistent signal output is of utmost importance for the system when utilized as a sensor, highlighting the critical aspect of surface reproducibility in determining sensor performance. Thus, employing the preliminary phase as the operational phase improves the replicability of the sensor surface and prevents long-term charge accumulation, thereby facilitating rapid detection. To achieve rapid detection of samples and ensure consistent results at the solid‒liquid interface, the initial stage is employed as the operational phase for the triboelectric sensor.

Figure 2d depicts the charge transfer process between the water droplet and the sensing polymer. When the water droplet falls and contacts the polymer surface, the droplet and polymer surface will be positively and negatively charged, respectively. Additionally, the continuous sliding of the droplet on the film surface results in the accumulation of positive charges on the droplet surface. As the water droplet slides toward the metal electrode position, the excess charge on the droplet induces a charge on the electrode. Simultaneously, the excess charge on the polymer film surface induces another charge after the water droplet moves away from the electrode position. This process repeats as the droplet slides across other surfaces of the sensing film until the water droplet eventually drops off the sensor. The corresponding voltage-time (V-t) response curve was obtained by monitoring the potential difference between the metal electrode and the ground environment during the process of droplet rolling.

A computational analysis was conducted using COMSOL 5.6 to simulate the potential liquid distribution during the motion of a droplet across two triboelectric sensors. The simulation results are shown in Fig. 2e. Initially, the droplet is assumed to be positively charged as a spherical ball. Upon contact between the droplet and the film, a charge transfer process occurs, causing the droplet to acquire a positive charge and the polymer film to acquire a complementary negative charge. As the sliding motion progresses, the charge on the droplet gradually intensifies. Subsequently, the presence of residual charge within the film creates charge attraction within the metal electrode through electrostatic induction, resulting in the generation of an induced current.

The theoretical model for charge distribution and transfer between solids and liquids was effectively described by the hybrid model proposed by Prof. Wang’s group, which considers the electric double layer (EDL) model based on the Gouy-Chapman-Stern theory^38–40^ and the “electron-cloud potential well” model^41^. Electron exchange is considered to be induced by the overlap of electron clouds of solid and liquid particles (atoms or molecules). Different materials and droplets possess distinct electronic affinities, which represent variations in their capability to gain or lose electrons. This factor influences the quantity of charge transferred from the droplets to the film per unit area^42^. Moreover, the varying hydrophobicity of materials influences the contact angle between the droplet and the film, resulting in diverse rates of change in the contact area over time^43^. The output waveforms of the TBIET represent the voltage response over time. With a constant external load, the signal strength is directly proportional to the total charge transferred per unit time^44^. This quantity is equivalent to the contact area multiplied by the charge transferred per unit area^43,45^.

To recognize different samples, we employ AI methods to analyze the overall signal variances, bypassing the need to establish accurate analytical models. Inspired by biomimicry, we used diverse film materials to detect a single droplet sample simultaneously, resulting in comprehensive signal changes. This multidimensional observation enhances the sample’s classification capability.

## Results and discussion

### Data preprocessing for the original data

**Fig. 3:**
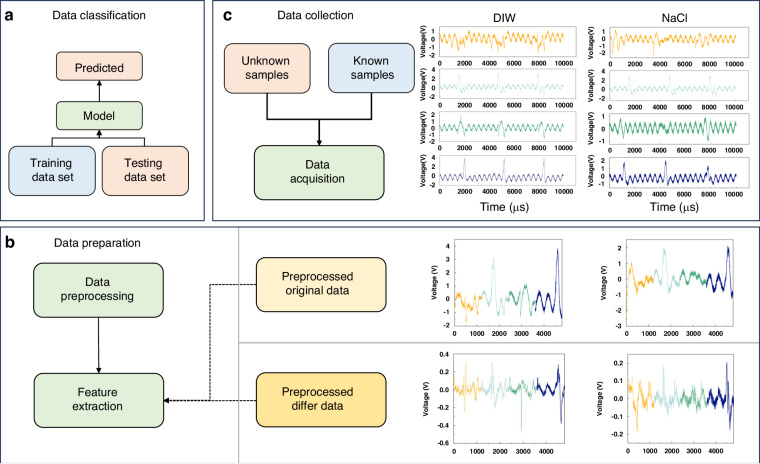
Process flow for data collection, preparation, and classification of the output signal of the TBIET device using the AI technique. **a** Data classification process, (**b**) data preparation process, (**c**) data collection process

Figure 3 shows our process flow diagram for the collection, preparation, and classification of the output signal of the TBIET device. The data obtained from the TBIET device are initially processed to extract relevant feature values. These feature values are then further classified using AI algorithms. To construct the model, it is necessary to collect a minimum of 30 samples for each category of data during the data collection phase. Figure 3b illustrates four distinct sets of unprocessed signals acquired from the initial three droplets captured by the triboelectric sensor array when exposed to solutions of deionized water (DIW) and sodium chloride (NaCl). Each peak in the figure corresponds to the flow of liquid sample droplets through the sensing polymer film, and any signal changes observed outside these peaks are attributed to system noise. Considering that the test environment remained unchanged throughout the experiment, the results of the data analysis are deemed reliable. Notably, the signal peak observed for DIW is greater in magnitude than that of NaCl, consistent with previous research findings on the triboelectric series^42^. The V-t response signals for all the samples are depicted in S3 and S5, exhibiting their characteristic behavior.

During the data analysis process, as shown in Fig. 3c, the initial step involves the identification of the local extremum within the dataset. Subsequently, the location of the time-domain signal in close proximity to the extremum is determined by considering its extremum position. The final analysis step includes the fusion of the 4-channel signals into eigenvalues. Notably, the flow regime in which droplets roll over the sensor array leads to distinct temporal positions of signal peaks across various channels. Thus, during the preliminary stage of data preparation, a single electrode is used to identify the local extremum, while the remaining channels derive feature values based on the selected time series without repeating the local extremum identification process.

The selection of suitable eigenvalues is of utmost importance when creating a predictive model that exhibits high classification performance and desirable robustness. To conduct a comprehensive evaluation of the output signal, we employed two feature extraction approaches to provide input values for the machine learning algorithm. The initial approach uses the original data (ORI) as feature values subsequent to the aforementioned data preparation procedure. The second approach computes the difference between the data acquired using the first approach and then applies window sweep filters to reduce noise. The outcome of these difference data (DIF) is commonly referred to as the eigenvalue, which can be mathematically expressed as follows:

1 $${Y}_{i}=\frac{1}{W}\mathop{\sum }\limits_{j=i-W+1}^{i}\left({X}_{j}-{X}_{j-1}\right)$$

where *W* represents the window size, which is set to 20; *Y* represents DIF data; and *X* represents ORI data. Both “*X*” and “*Y*” are time series data.

### AI results for different types of liquid samples

#### Results of LDA analysis

First, the LDA method is employed to visualize the extracted feature values. LDA is a well-established classic supervised dimensionality reduction method that has been widely used in the field of electronic tongue systems^46,47^. LDA aims to maximize the difference between classes by decreasing the intraclass dissimilarity. The detailed calculation process and formula of LDA are outlined in S2.

**Fig. 4:**
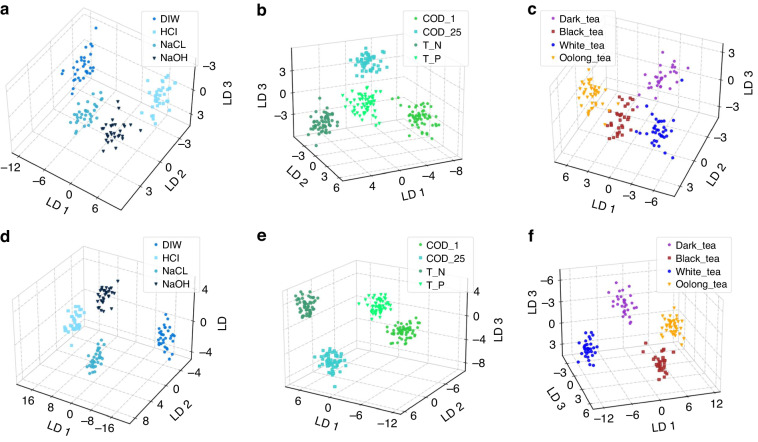
The LDA dimensionality reduction visualization results for three types of samples. **a** Basic chemical samples with ORI feature values. **b** Environmental samples with ORI feature values. **c** Food (tea) samples with ORI feature values. **d** Basic chemical samples with DIF feature values. **e** Environmental samples with DIF feature values. **f** Food (tea) samples with DIF feature values

Figure 4a–c presents the LDA outcomes using the feature values of ORIs for chemical, environmental, and food samples. The results of LDA analysis using DIF feature values for the aforementioned samples are shown in Fig. 4d–f. Regardless of the values assigned to the features, the samples are classified into four distinct zones in the context of chemical and environmental analysis. In the case of food samples (e.g., tea), there is a small area of overlap between black tea and oolong tea when the ORI feature values are used. However, when utilizing DIF feature values, these samples are further categorized into four distinct regions. The obtained visualization results of the LDA algorithm demonstrate the efficacy of the TBIET device in accurately classifying chemical, environmental, and food samples.

#### SVC and RF classification results

The results of the LDA algorithm technique demonstrate that the investigated samples can be classified, although this technique provides only approximate classification data. To achieve more precise results, we apply two well-established machine learning algorithms, SVM and RF, for in-depth analysis. Figure 3a shows the corresponding process of data classification. First, the data are split into two parts, with one part designed as the training set. In this set, the sample labels are retained for model training by machine learning algorithms. The remaining data are assigned to the test set, assuming that the sample labels are unknown and can be predicted by the trained model. Finally, the predicted labels are compared with the actual labels of the test set to assess the precision of the model.

The leave-one-out approach (LOO) was employed to partition the data. In this approach, one data point is reserved for testing, while the remaining data points are utilized for training. The test data points are then successively shifted across the entire dataset. The final classification result is determined by calculating the average of all test samples. Given the nature of this task, the support vector classification (SVC) approach of SVM is utilized to address the multiclassification problem. The fundamental principle underlying the SVM approach is the identification of a hyperplane between samples. In contrast, the RF method focuses on averaging individual leaf nodes. The detailed procedures and formulas for the SVM and RF approaches are given in S2.

**Fig. 5:**
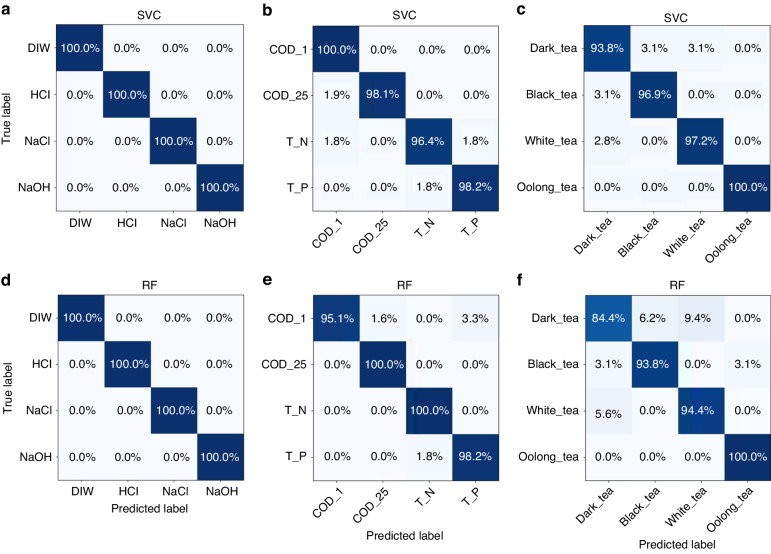
Confusion matrix of SVC and RF classification results with ORI feature values for three sample types. **a** SVC classification results for chemical samples. **b** SVC classification results for environmental samples. **c** SVC classification results for food (tea) samples. **d** RF classification results for chemical samples. **e** RF classification results for environmental samples. **f** RF classification results for food (tea) samples

The evaluation of the classification outcomes of these two algorithms is conducted using confusion matrices, as shown in Fig. 5 and Fig. S3 (in S4). The confusion matrix is a tabular representation where the predicted labels are represented by rows and the true labels are represented by columns. A sample is considered to be accurately predicted when the label predicted by the model aligns with the true label. Thus, the elements located along the diagonal of the matrix correspond to the fraction of samples that were accurately predicted. The classification outcomes of the SVC and RF algorithms with ORIs as the feature values are presented in Fig. 5. The results indicate that all chemical samples were predicted correctly by both algorithms. For environmental samples, both algorithms achieved high classification accuracy, with a correct prediction rate (CPR) per sample exceeding 95.1%. In the case of food samples, excluding the CPR of dark tea (which was 84.4% when using the RF algorithm), the CPRs of other types of tea samples exceeded 93.8%. The classification results of SVC and RF with DIF as feature values are provided in S4. The findings indicate that the accurate prediction of all chemical samples was achieved, regardless of the method employed.

The CPR of the RF algorithm for COD-25% in environmental samples was found to be 84.9%. In contrast, the CPR of the RF method for other types of samples was greater than 92.7%. Compared with the RF algorithm, the SVC algorithm achieved greater classification accuracy. Specifically, the SVC approach attained a classification accuracy rate of over 96.2% for all samples. The CPR for all food samples, with the exception of black teas, exceeded 93.8%. Notably, the RF algorithm misclassified 12.5% of the black teas.

The metric of classification accuracy (ACC) denotes the ratio of correctly predicted samples to the overall number of samples, as listed in Table 1. In the context of environmental data, when employing the ORI as the eigenvalue, the SVM algorithm exhibits little ACC compared to the RF approach. Specifically, the ACCs achieved by the SVM and RF algorithms are 98.2% and 98.3%, respectively. In other cases, the accuracy of the SVM algorithm is greater than or equal to the accuracy of the RF approach.

**Table 1:** Classification accuracy for the three types of samples

Feature values	Chemical samples		Environmental samples		Food samples	
	SVC	RF	SVC	RF	SVC	RF
ORI	100.0	100.0	98.2	98.3	97.0	93.2
DIF	100.0	100.0	97.3	92.7	96.3	93.3

Additionally, the classification accuracy of individual electrodes for these samples is presented in Table S1. Among the 48 classification results, only five individual electrode results slightly exceeded the final output results. Notably, these instances were related to measurements of environmental samples using PE and PDMS, as well as tea samples using PTFE. In all other cases, the classification accuracy of the individual electrodes was significantly lower than that achieved after the fusion of the four electrodes.

Overall, the ACC in all experimental cases was consistently greater than 92.7%. These robust classification outcomes are consistent with the visual representations of the LDA algorithm, as depicted in Fig. 4. These findings confirm that the TBIET device exhibits proficient classification capabilities across chemical, environmental, and food sample scenarios.

### AI results for the same sample at different concentrations

**Fig. 6:**
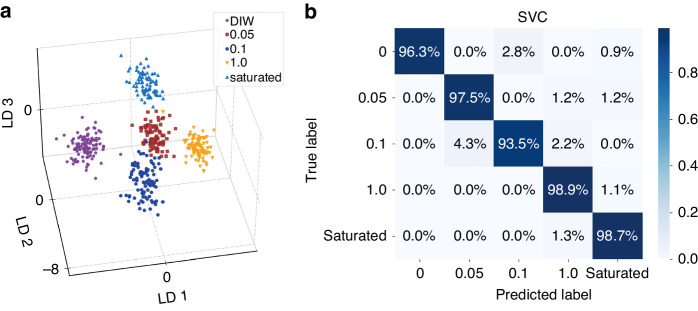
Classification of the ORIs of NaCl solutions at five different concentrations. **a** LDA visualization of dimensionality reduction. **b** Confusion matrix of the SVC results

To demonstrate the classification capability of the TBIET system for the same sample at various concentrations, NaCl solutions with concentrations of 0 (DIW), 0.05, 0.1, 1.0, and 5.4 (saturated) mol/L were tested. The LDA and SVM classification results utilizing the ORI feature values are shown in Fig. 6, while the RF method results are presented in Fig. S4. In the LDA plot, the five sample classes distinctly occupy distinct regions. When employing SVC for classification, excluding the 0.1 mol/L sample (which was accurately classified at a rate of 93.5%), the proportions of correctly classified samples for all other concentrations exceeded 96.3%. When utilizing RF classification, all sample types were correctly classified at a rate exceeding 94.4%. Furthermore, the LDA, SVM, and RF results obtained using DIF as the feature value are provided in Figs. S6 and S7. The DIF feature value still proves effective when classifying the samples, and the SVM and RF classifiers achieved accuracy rates exceeding 88.9% and 93.5%, respectively.

Moreover, a comparison of the ACCs for the individual electrodes and the combined electrodes is shown in Table S2. Crucially, in contrast to the results of the different types of samples presented in Table S1, all eight results exhibited accuracies noticeably lower than the ultimate classification accuracy. The individual electrode accuracy ranged from 43.5% to 85.1%, markedly below the aggregated accuracy exceeding 92.9%.

These findings highlight the efficacy of the TBIET system in categorizing varying concentrations of the same solution. The fusion of multiple channels significantly increased the classification accuracy.

## Discussion

We combined triboelectric sensing arrays and AI to develop a portable, self-powered liquid sensor with broad-spectrum responsiveness. Table 2 presents a comparison between our EBIET system and a standard E-tongue system, highlighting the differences in sensor types. Traditional E-tongue functions are based on different sensor principles, including potentiometry, voltammetry, optical addressing, and impedance spectroscopy^48^. The sensing array of the potentiometry E-tongue comprises a working electrode array and an Ag/AgCl reference electrode. The structure of the working electrode comprises different types of lipid membranes. During the experiments, the electrical potential on the surface of the lipid membrane was measured and compared to the potential of the reference electrode. The voltammetry sensing array utilized a three-electrode configuration, consisting of an Ag/AgCl reference electrode, a working electrode array, and a counter electrode. The working and counter electrodes are commonly fabricated using stable metals such as silver, titanium, gold, and platinum. Optical addressing sensors utilize different color-developing agents that react with metal ions in solution for color development. Additionally, it incorporates E-tongue technology, which is based on the principles of stripping voltammetry. An impedance spectrum sensor is usually composed of ultrathin films that are fabricated using different materials deposited on interdigital electrodes. In comparison to the aforementioned E-tongues, TBIET offers the most convenient structure and the most accessible sensing material because it only requires a simple process of attaching commercial dielectric films onto metal electrodes. In addition, EBIET offers the advantages of simple operation and rapid response time. Furthermore, unlike the E-tongues that necessitate the immersion of electrodes in the droplets, the EBIET sloley requires the identification of the droplets, rendering it an almost non-invasive detection method.

**Table 2:** Comparison of EBIET and another E-tongue system

Sensor type	Electrode material	Sample volume	Response principle	Merits or drawbacks	Reference
Triboelectric sensor	Dielectric Film	3 μl	Triboelectric Characteristics & Flow State	Convenient structure, Easy to operate, Nearly non-invasive, Self-powered, Detection ability of flow state	This work
Voltammetric sensor	Rare Metals	15 mL	Electrochemical Characteristics	Expensive electrode materials, Easy to operate	^9,52^
Optical addressing sensor	Polymers Nanoparticles	1 mL	Electrochemical Characteristics	Complex structure, Difficult to operate	^53^
Impedance spectrum sensor	Ultrathin Langmuire-Blodegett Film	15 mL	Electrochemical Characteristics	Expensive electrode materials, Easy to operate	^54,55^
Potentiometric sensor	Conductive Polymer Film Phthalocyanine Film Plasticized Polymer Polycrystalline	15 mL	Electron Exchange & Hydrophobicity	Poor stability of the electrode material, Easy to operate	^2^

The detecting capabilities of an E-tongue are considerably influenced by the physical or chemical information present in response signals. The detecting capabilities of the E-tongue increase in proportion to the number of mechanisms incorporated in the response signals. The conventional E-tongue is capable of identifying the electrochemical characteristics of the samples, such as their chemical potential (related to electron exchange ability), the diffusion capacity of specific particles within the sample, and the double layer phenomenon, which is often explained using the Gouy‒Chapman–Stern model^39,40^. The impact of the electron exchange ability^38^ and surface double layer^40^ on the response signals of TBIET has been demonstrated. The EBIET device stands out as the sole electronic tongue (E-tongue) capable of detecting signals emitted by flowing droplets. The distribution of surface charges on droplets is influenced by various flow states, leading to modifications in the response signals^42,43^. Thus, the TEBIET is distinguished as the sole E-tongue platform with a response signal that is subject to the impact of the flow characteristics of the liquid. Furthermore, compared to the most recent triboelectric E-tongue^34^, we adopted a biomimetic approach that uses different materials to mimic the various taste buds on the human tongue^1^ and constructed an E-tongue system. Our experimental results indicate that the multielectrode fusion system exhibits superior classification capability in comparison to systems using a single sensitive material. Moreover, the enhancement in the classification ability became more pronounced in the multielectrode system as the sample compositions became more similar.

In summary, the TBIET system exhibits a highly convenient structure, possesses practically non-invasive qualities, demonstrates self-powering capabilities, and provides response signals that encompass a wide variety of mechanistic information. This novel sensor has the potential to broaden the scope of application for the initial electronic tongue utilized in laboratory settings.

The successful classification of chemical samples based on ion samples supports the potential applicability of utilizing groundwater sample analysis for mineral exploration purposes. In the analysis of environmental samples, we successfully classified varying quantities of COD, thereby indicating the potential for realizing a real-time and on-site monitoring system for tracking environmental conditions. Moreover, the achievement of effective categorization demonstrated the feasibility of implementing the TBIET in agricultural fields, specifically for establishing an internet-connected, self-sustaining monitoring system. Such a system would enable real-time tracking of plant development as well as identification and management of pest and disease infestations throughout the growth cycle. In addition, it is possible to optimize the design of TBIET to facilitate the identification of human bodily fluids, such as urine and blood, for the early diagnosis of certain medical diseases.

To establish the TBIET for commercial applications, it is necessary to overcome some current limitations. The primary concern is that TBIET is dependent on the accurate identification of spectra and requires a detection environment that is highly stable and capable of producing consistent results. Nevertheless, even when considering droplets of the same type, the contact states with the triboelectric sensing film might vary, including different locations, angles, and velocities. Consequently, these variations can lead to distinct spectra. The presence of variability in the data can potentially result in misidentification by the system, thus presenting a significant obstacle in attaining outcomes that are both consistent and dependable. To address this challenge, a possible approach involves integrating the microfluidic system with TBIET to regulate the contact parameters between the droplet and the triboelectric sensing film. This integration would reduce the influence of the aforementioned variations on the resulting spectra. Consequently, the enhancement of the sensor’s responsiveness leads to increased consistency and repeatability. An alternative approach includes extensive data collection from real-world scenarios. By systematically collecting a diversified array of data across different contact states, it is possible to develop a comprehensive and diverse database. Such a database can be combined with advanced deep learning models, such as convolutional neural network (CNN)^49^ and transformer^50^ algorithms, to improve the classification accuracy of the system and expand its range of applications. These data-driven techniques can help establish robust models capable of accurately identifying and classifying liquids by analyzing their spectral characteristics. Moreover, in real-world scenario applications, when dealing with interference from unknown samples, to enhance the specificity of recognition, considering classification as open-state recognition and choosing appropriate methods can be beneficial^51^.

## Conclusion

In this paper, we presented a triboelectric bioinspired E-tongue that combines liquid‒solid power generation with artificial intelligence to realize an artificial taste-sensing device. The device integrates four different triboelectric polymer films for sensing four series of droplets on a single glass chip. The performance of the TBIET was evaluated using three types of liquid materials, including chemical, environmental, and food samples. Additionally, liquid samples with identical solute compositions but varying concentrations were also examined to assess the system’s classification capabilities at different concentrations. Signal analysis was performed utilizing the LDA, SVC, and RF algorithms. The experimental findings demonstrated a classification accuracy exceeding 92.7% across all categories. Through careful selection of an optimal algorithm, we achieved a classification accuracy of 100% for ion-type DIW, HCl, NaOH, and NaCl in chemical solutions. The device attained a high accuracy of 98.2% in identifying chemical oxygen demand (COD) and total nitrogen (T-N) in environmental samples characterized by concentrations as low as 1 mg/L. The device demonstrated a classification accuracy of 97.0% for identifying four prevalent varieties, including white, black, dark, and oolong teas, and an accuracy of 96.9% for the classification of NaCl solutions with five different concentrations. The results preselected in this study indicate that the TBIET device exhibits a high level of accuracy in classifying data, thus confirming its potential suitability for use in the specified scenarios. This self-powered and portable detection device represents a significant advancement in liquid sensor technology, offering a versatile and practical solution for E-tongue applications.

### Supplementary information

Supplementary Information

## Data Availability

Data will be made available on request.
