# Supplementary material for: Bioinspired integrated triboelectric electronic tongue
Source: Microsyst Nanoeng. 2024 May 8;10:57. doi: 10.1038/s41378-024-00690-9 (PMC11079038; doi:10.1038/s41378-024-00690-9)
Supplement: Supplementary file 1 — Supplementary Information [file 41378_2024_690_MOESM1_ESM.docx]

**Supporting information**

**Bioinspired integrated triboelectric electronic tongue**

Jiaming Liu^a^*, Jingui Qian^a^*^#^, Adil Murtazt^b^, Yali Bi^a^, Haoyi Wu^b^, Xuefeng Hu^a^, Zuankai Wang^#c^, and Wei Zhang^b#^

^a^Anhui Province Key Laboratory of Measuring Theory and Precision Instruments, School of Instrumental Science and Optoelectronics Engineering, Hefei University of Technology, Hefei 230009, Anhui, China.

^b^School of Physics and Optoelectronic Engineering, Guangdong University of Technology, Guangzhou 510006, Guangdong, China.

^c^Department of Mechanical Engineering, The Hong Kong Polytechnical University, Hong Kong SAR, China.

*These authors contributed equally to this work.

^#^To whom all correspondence should be addressed.

E-mail addresses: [zhangw0520@gdut.edu.cn](mailto:zhangw0520@gdut.edu.cn), [zk.wang@polyu.edu.hk](mailto:zk.wang@polyu.edu.hk), [jgqian@hfut.edu.cn](mailto:jgqian@hfut.edu.cn)

**S1. Optical image of the fabricated device and assembled system**


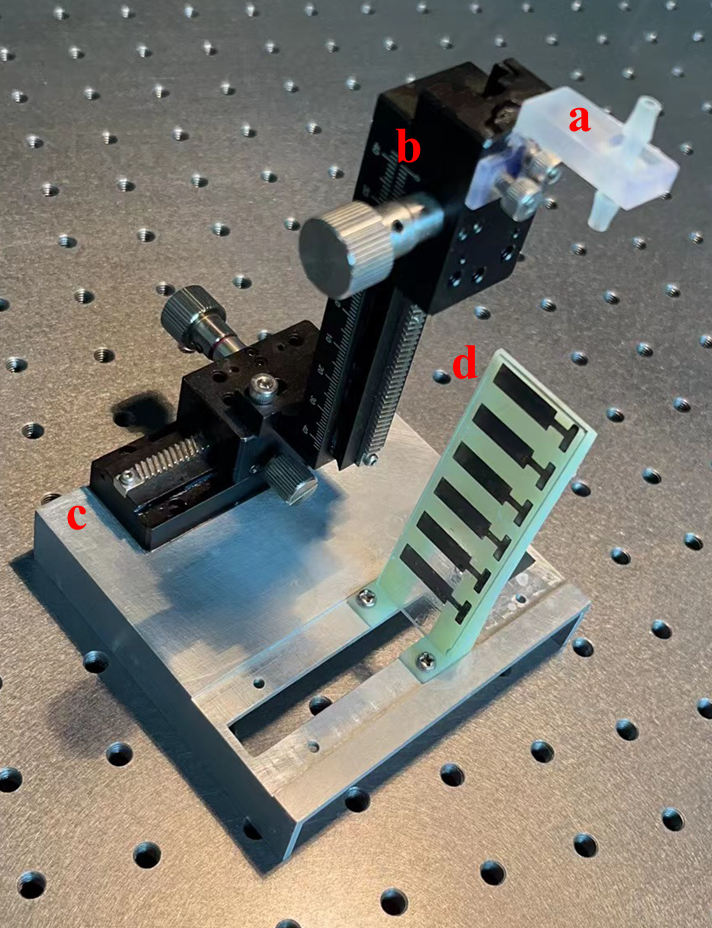


Fig.S1 A multifunction platform for performative characterization of TBIET. (a) A dropper retention bracket. (b) A vertical and horizontal adjustable slider. (c) A metal base for fixing setups. (d) A Fabricated and assembled TBIET device.

**S2. Process of LDA SVM and RF algorithm**

LDA (Linear Discriminant Analysis) is a dimensionality reduction technique used for classification tasks. Assuming there are C classes, each class's data follows a normal distribution, with mean vectors denoted by μ and covariance matrices denoted by Σ. The goal of LDA is to find a projection matrix W that maximizes the between-class scatter and minimizes the within-class scatter, which can be expressed as follows:

$J\left( W \right)=\frac{W^{T}\times\sum b\times W}{W^{T}\times\sum w\times W}$ (i)

where Σb is the between-class scatter matrix and Σw is the within-class scatter matrix, and are defined as follows:

$\sum b=\sum t-\sum w$ (ii)

$\sum t=\frac{1}{N}\sum_{i=1}^{N} \left[ \left( x_{i}-\mu_{i} \right)\times\left( x_{i}-\mu_{i} \right)^{T} \right]$ (iii)

$\sum w=\frac{1}{N}\sum_{i=1}^{N} \sum_{j=1}^{M} \left[ \left( x_{ij}-\mu_{i} \right)\times\left( x_{ij}-\mu_{i} \right)^{T} \right]$ (iv)

N is the total number of samples, M is the dimensionality of the data, and $x_{i}$ and $x_{ij}$ denote the ith sample and jth feature of the data, respectively. The mean vector of each class is denoted by $\mu_{i}$.

SVC (Support Vector Classification) is a type of Support Vector Machine (SVM) algorithm that is commonly used in multi-classification situations. SVM maps vectors into a higher-dimensional space where a maximum interval hyperplane is established. Two hyperplanes parallel to each other are built on either side of the hyperplane separating the data, and separating the hyperplane maximizes the distance between the two parallel hyperplanes. It is assumed that the greater the distance or gap between parallel hyperplanes, the smaller the total error of the classifier. where the 0-1 function is the number of mids-divided samples, and where is the feature mapping function. For linearly separable support vector machines, solving the problem can be transformed into an optimization solution problem with constraints.

The formula for the SVM algorithm can be expressed as follows:

${min}_{w,b,\varepsilon} {\frac{1}{2}\left\| \omega\right\|}^{2}+C\sum_{i=1}^{N} \varepsilon_{i}$ (v)

subject to:

$y_{i}\left( \omega^{T}\varphi\left( x_{i} \right)+b \right)\geq1-\varepsilon_{i},i=1,2,\ldots,N$ (vi)

$\varepsilon_{i}\geq0,i=1,2,\ldots,N$ (vii)

Where$\omega^{T}$ is the weight vector, b is the bias, $\varphi\left( x_{i} \right)$ is the feature vector of the $\varepsilon_{i}$ data point, $y_{i}$ is the corresponding label, $x_{i}$ is the slack variable, and C is the regularization parameter. The objective function minimizes the norm of the weight vector $\left\| \omega\right\|^{2}$ while balancing the trade-off between the margin and the number of misclassified data points (represented by the sum of slack variables). The constraints ensure that all data points are classified correctly or with a certain tolerance given by $x_{i}$.

The random forest (RF) algorithm works by randomly building a forest of decision trees, each of which is independent of the others. When a new input sample is introduced, each decision tree in the forest makes a separate judgment to determine the class to which the sample belongs (for the classification algorithm). The forest then determines which class was selected most often and predicts that as the class for the sample. Here is the formula for the RF algorithm:

$f\left( x \right)=\frac{1}{T}\sum_{i=1}^{T} f_{i}\left( x \right)$ (viii)

$f_{i}\left( x \right)=\sum_{j=1}^{m} c_{ij}I\left( x\in R_{ij} \right)$ (ix)

where $f_{i}\left( x \right)$ represents the prediction result of the i-th decision tree, T represents the number of the decision tree, m represents the number of leaf nodes in the decision tree, $c_{ij}$ represents the class corresponding to the j-th leaf node in the i-th decision tree, and $R_{ij}$ represents the sample space region covered by the j-th leaf node in the i-th decision tree.

**S3.** **Characteristic peak of the V-t response signal for different types of samples**


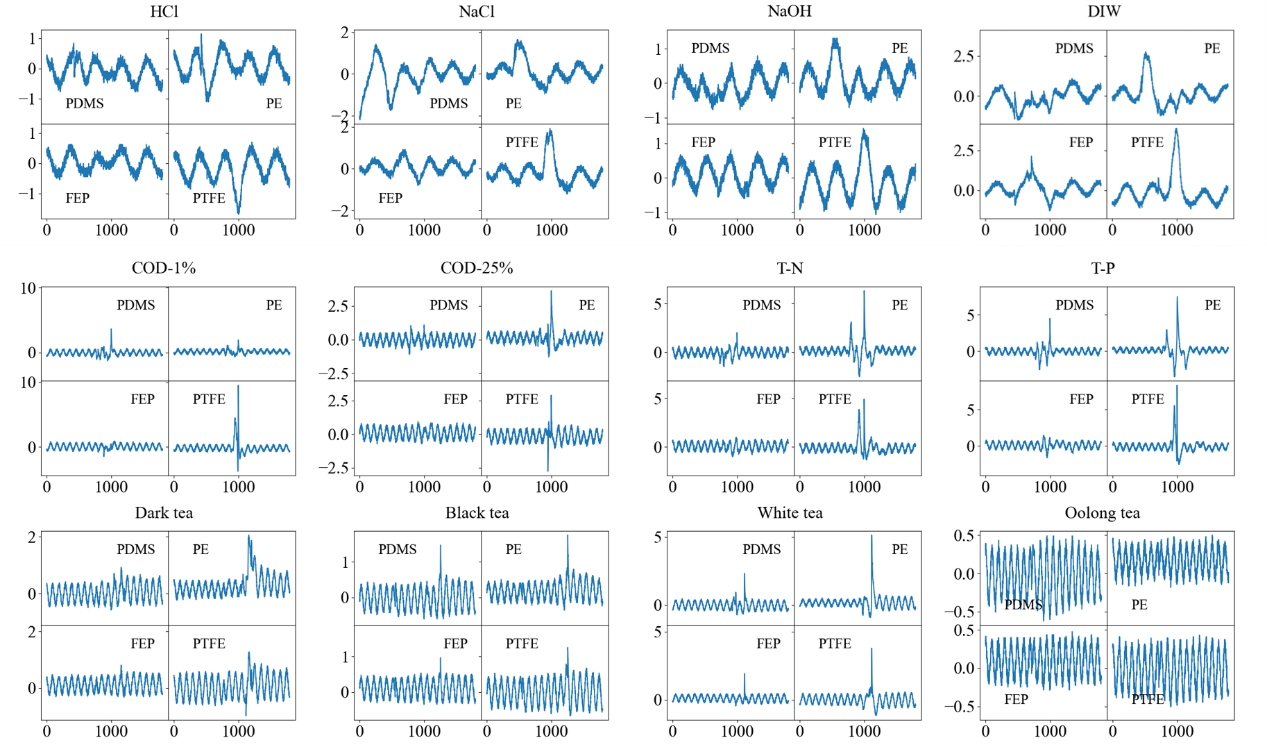


Fig.S2 One characteristic peak of the V-t response signal for all samples

**S4. Result of SVM and RF algorithm with DIF feature values**


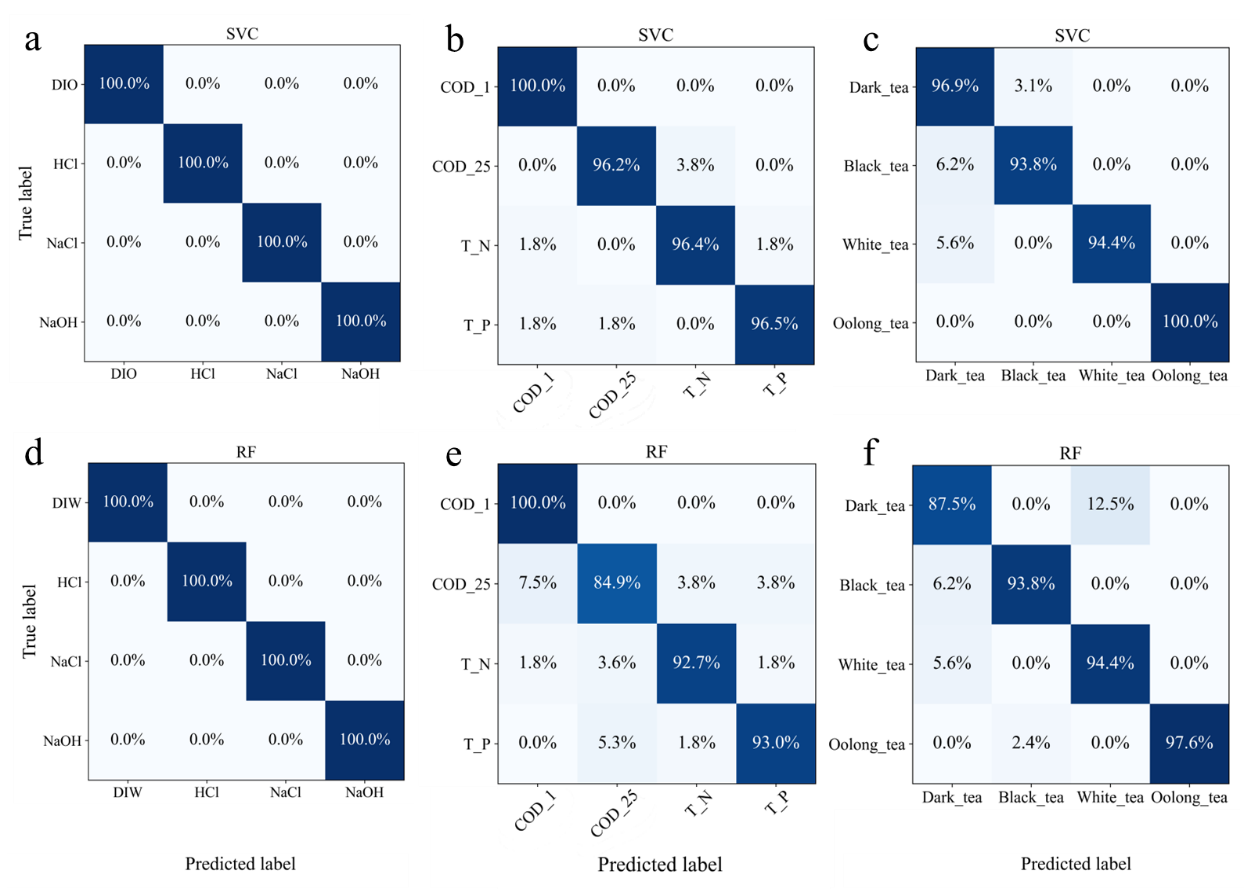


Fig.S3 Confusion matrix of SVC and RF classification results with DIF feature values for three sample types. (a) SVC classification results for chemical samples. (b) SVC classification results for environmental samples. (c) SVC classification results for food (tea) samples. (d) RF classification results for chemical samples. (e) RF classification results for environmental samples. (f) RF classification results for food (tea) samples.

**Table S1 Comparison of accuracy (ACC) between individual electrodes and the combined electrodes for different type of samples.**

| **Feature Values** | **channel** | **Chemical samples** | | **Environmental samples** | | | | **Food Samples** | |
| --- | --- | --- | --- | --- | --- | --- | --- | --- | --- |
|  |  | SVM | RF | | SVM | RF | SVM | | RF |
| ORI | **all** | **100.0** | **100.0** | | **98.2** | **98.3** | **97.0** | | **93.2** |
|  | PDMS | 99.2 | 96.9 | | 98.7 | 94.6 | 83.1 | | 77.5 |
|  | PE | 100.0 | 96.9 | | 98.7 | 96.4 | 93.7 | | 90.8 |
|  | FEP | 98.5 | 94.6 | | 93.7 | 80.3 | 87.3 | | 70.4 |
|  | PTFE | 100.0 | 98.4 | | 86.1 | 87.9 | 92.3 | | 94.3 |
| DIF | **all** | **100.0** | **100.0** | | **97.3** | **92.7** | **96.3** | | **93.3** |
|  | PDMS | 99.2 | 87.7 | | 95.6 | 93.7 | 73.9 | | 78.9 |
|  | PE | 99.2 | 87.7 | | 96.4 | 95.5 | 88.7 | | 92.3 |
|  | FEP | 96.9 | 86.9 | | 88.8 | 78.5 | 78.9 | | 78.1 |
|  | PTFE | 100.0 | 98.4 | | 83.9 | 88.3 | 91.5 | | 89.4 |

S5 **Characteristic peak of the V-t response signal for** **same types of samples with different concentrations**


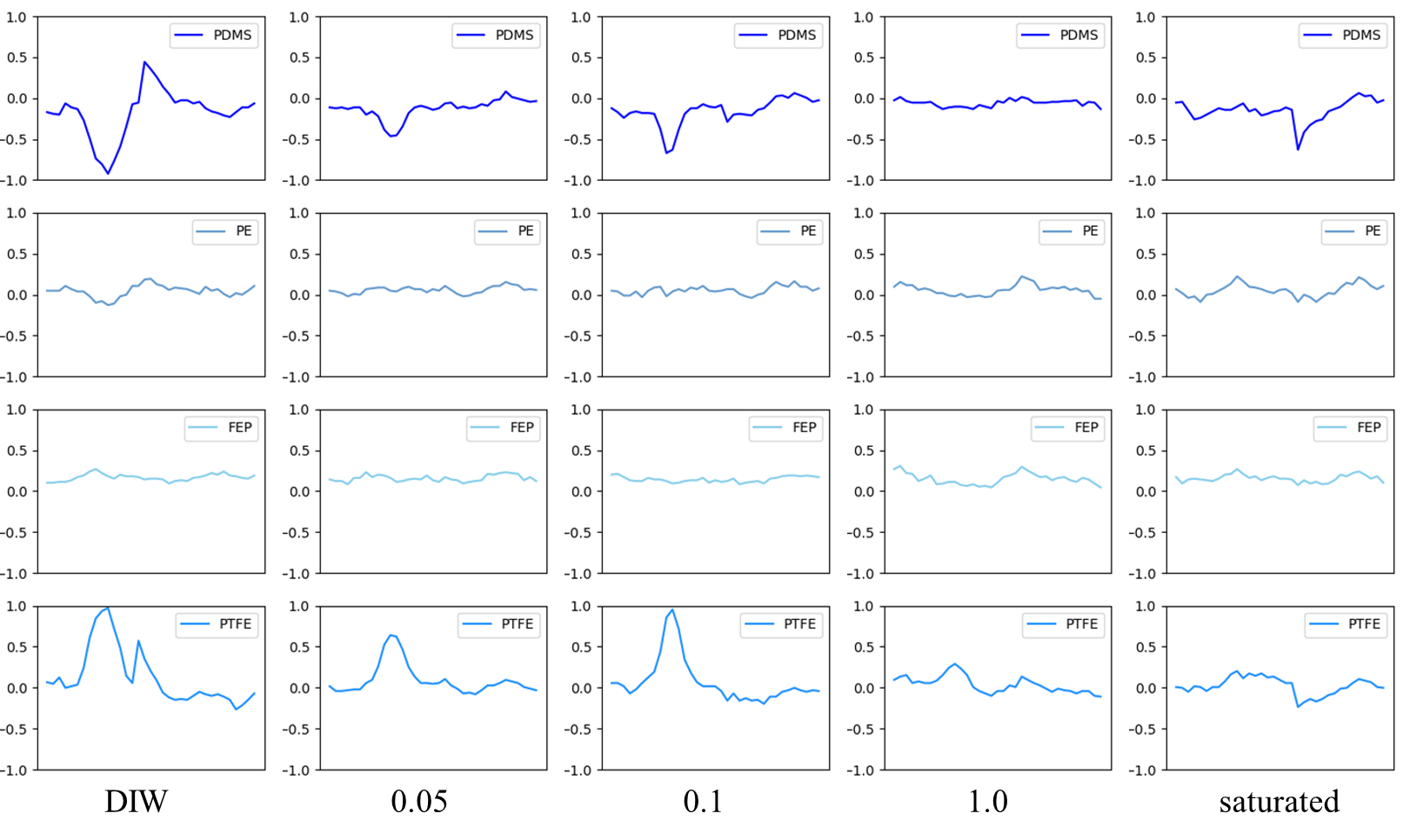


Fig. S4 One characteristic peak of the V-t response signal for samples with different concentrations

**S6. AI Result for same types of samples with different concentrations**


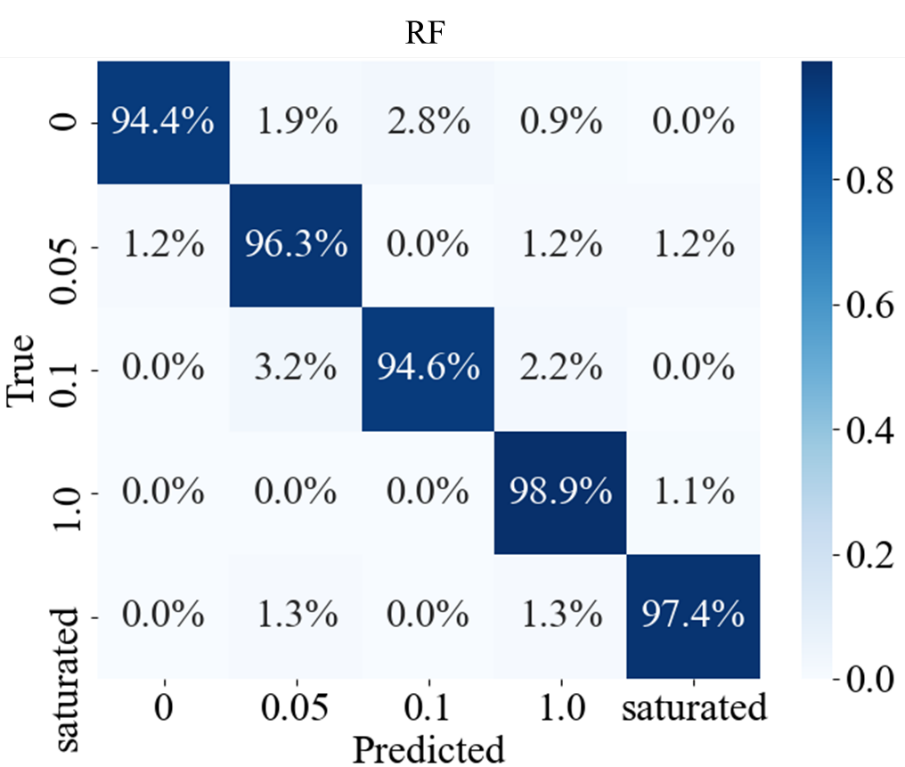


Fig. S5 RF classification results using ORI feature value for NaCl solution with different concentrations


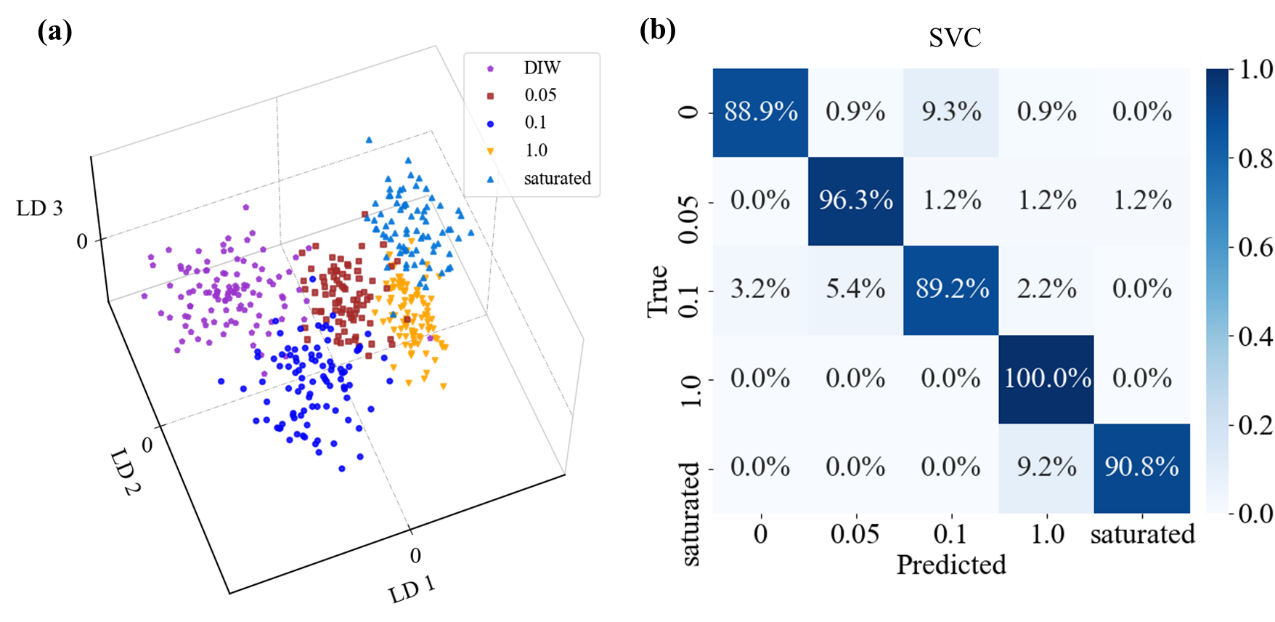


Fig. S6 LDA and SVC result using DIF feature value for NaCl solution with Different Concentrations


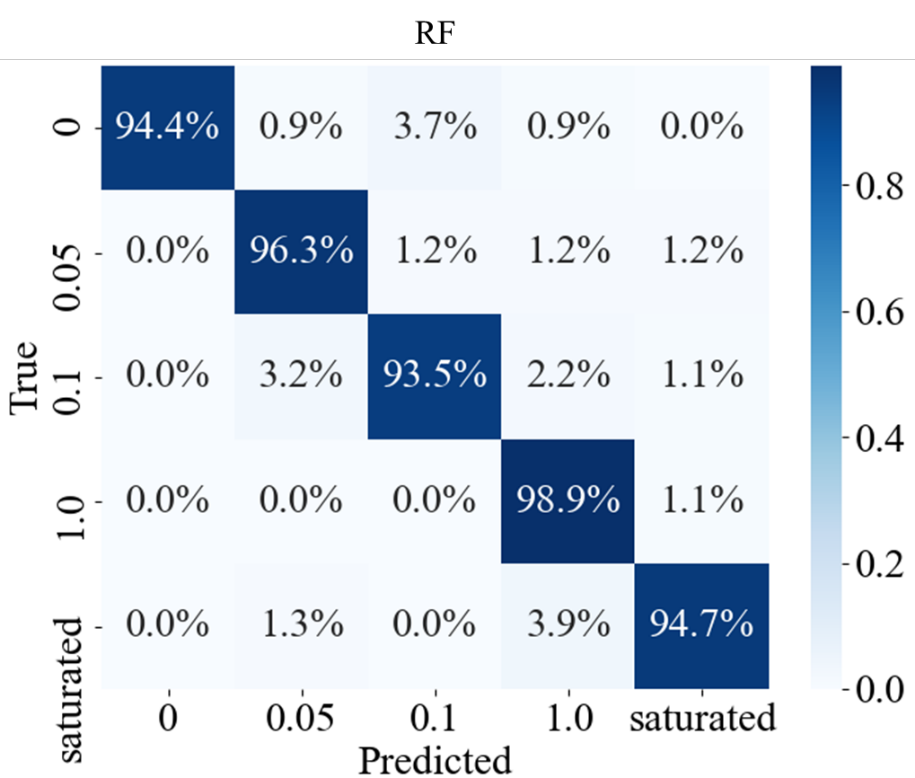


Fig. S7 RF classification results using DIF feature value for NaCl solution with different concentrations

**Table S2: Comparison of accuracy (ACC) between individual electrodes and the combined electrodes for NaCl solution samples with different concentrations.**

| Feature Value |  | SVM | RF | Feature  Value |  | SVM | RF |
| --- | --- | --- | --- | --- | --- | --- | --- |
| ORI | **all** | **96.9** | **96.2** | DIF | **all** | **92.9** | **95.6** |
|  | PDMS | 84.7 | 85.1 |  | PDMS | 78.7 | 76.9 |
|  | PE | 43.5 | 70.5 |  | PE | 62.1 | 60.5 |
|  | FEP | 54.3 | 78.5 |  | FEP | 74.3 | 71.0 |
|  | PTFE | 76.7 | 85.6 |  | PTFE | 75.2 | 74.9 |
